# Utilization of Incense Stick Ash in Hydrometallurgy Methods for Extracting Oxides of Fe, Al, Si, and Ca

**DOI:** 10.3390/ma15051879

**Published:** 2022-03-03

**Authors:** Virendra Kumar Yadav, Govindhan Gnanamoorthy, Krishna Kumar Yadav, Ismat H. Ali, Abdulaziz A. Bagabas, Nisha Choudhary, Shalini Yadav, Rajendran Suriyaprabha, Saiful Islam, Shreya Modi, Marina Cabral-Pinto

**Affiliations:** 1 Department of Microbiology, School of Sciences, P P Savani University, Kosamba, Surat 394125, Gujarat, India; 2 Department of Inorganic Chemistry, University of Madras, Guindy Campus, Chennai 600025, Tamil Nadu, India; gnanadrdo@gmail.com; 3 Faculty of Science and Technology, Madhyanchal Professional University, Ratibad, Bhopal 462044, Madhya Pradesh, India; envirokrishna@gmail.com; 4 Department of Chemistry, College of Science, King Khalid University, P.O. Box 9004, Abha 61413, Saudi Arabia; ihali@kku.edu.sa; 5 National Petrochemical Technology Center (NPTC), Materials Science Research Institute (MSRI), King Abdulaziz City for Science Technology (KACST), P.O. Box 6086, Riyadh 11442, Saudi Arabia; abagabas@hotmail.com; 6 Department of Environmental Sciences, School of Sciences, P P Savani University, Kosamba, Surat 394125, Gujarat, India; nishanaseer03@gmail.com; 7 Department of Civil Engineering, Rabindranath Tagore University, Raisen 462045, Madhya Pradesh, India; shaliniy2000@gmail.com; 8 School of Nanosciences, Central University of Gujarat, Gandhinagar 382030, Gujarat, India; sooriyarajendran@gmail.com; 9 Civil Engineering Department, College of Engineering, King Khalid University, Abha 61413, Saudi Arabia; sfakrul@kku.edu.sa; 10 Department of Microbiology, Shri Sarvajanik Science College, Mehasana 384001, Gujarat, India; shreyamodi20@yahoo.in; 11 Geobiotec Research Centre, Department of Geoscience, University of Aveiro, 3810-193 Aveiro, Portugal

**Keywords:** incense stick ash, calcite, ferrous minerals, silica, alumina, zeolites

## Abstract

With rapid industrialization, there is an ever-increasing demand for iron oxides, calcium oxides, aluminum oxides, silica, and zeolites as raw materials for various industries, but reserves of such metal oxides are continuously diminishing. Therefore, there is an urgent need to explore new alternatives for such value-added minerals. One such material is incense stick ash (ISA), which is among the most unexplored byproducts from residential and holy places. Currently, ISA is of no use and it is disposed of in millions of tons (MTs) in rivers and other water bodies in India due to its sacred value. The major chemical composition of ISA is calcium, silica, alumina, ferrous minerals, magnesium, and traces of Na, K, P, Ti, etc. Major fractions of ISA, i.e., 50–60%, are made up of calcium and magnesium oxides; 20–30% of ISA is made up of silica, alumina, and ferrous minerals, as revealed by X-ray fluorescence spectroscopy (XRF). In the present research work, methods of recovery of value-added micro and nano minerals from ISA are suggested, using cost-effective techniques and an eco-friendly approach. Firstly, magnetic fractions were recovered by a magnetic separation method; then, alumina, silica, and calcium oxides were synthesized from non-magnetic fractions. The confirmation of the synthesized and extracted nanomaterials was done by Fourier transform infrared spectroscopy (FTIR), particle size analyzer (PSA), X-ray diffraction (XRD), field emission scanning electron microscopy with electron diffraction spectroscopy (FESEM-EDS), and transmission electron microscopy (TEM). The purity of synthesized particles varied from 40–80%. In the future, ISA will prove to be an alternative resource material for Fe, Ca, Si, C, Al, and zeolites, which will minimize solid waste pollution and water pollution arising due to the disposal of ISA into water bodies.

## 1. Introduction

Incense sticks are long cylindrical-shaped fragrant materials used for spreading aroma in homes and temples during the worshipping of deities, and are sometimes also used as an insect-repelling agent [1,2]. The burning of incense sticks leaves behind ashes known as incense stick ash (ISA) [3,4,5,6]. Every year, a million tons (MTs) of incense sticks are burned around the whole globe, out of which India alone is expected to have had a turnover of around Rs 7500–8000 crore from August to December 2021. During April–September 2020, India generated revenues of about Rs 346.52 crore by exporting incense and incense sticks to 150 countries around the globe [3]. The burning of incense sticks is more popular in Buddhism and Hinduism, so most incense sticks are consumed in South Asian countries like Taiwan, China, Japan, India, etc. [7]. India, being the third-largest producer of incense sticks, generates a huge amount of ISA, which is mainly disposed of into rivers due to the sacred value of ISA [4]. To date, ISA has been used for the recovery of ferrous particles and their further processing for the synthesis of crystalline and amorphous iron oxide nanoparticles. Besides this, ISA has also been used for the synthesis of zeolites rich in calcium as reported by Yadav et al., 2021. Furthermore, Jain et al., instead of using any specific mineral of ISA, have used ISA as such for the removal of Victoria blue dye from wastewater [8]. ISA has gained attention as an economical adsorbent due to its highly porous nature. There is an alarming issue in terms of environmental water pollution due to the presence of numerous toxic heavy metals and a high amount of alkali metals in Indian ISA [9,10]. Once this heavy metal- and alkali metal-loaded ISA is disposed into the water, it may lead to water pollution. This increases the hardness of the water, and a higher level of heavy metals may pose a potential threat to the living beings in the aquatic system [11]. Therefore, it is of the utmost necessity to explore value-added applications of ISA in order to protect our environment from the pollution arising from ISA disposal. In order to minimize the toxicity of ISA, Yadav et al., 2021 reported sequential approaches for the transformation of hazardous ISA into less toxic value-added minerals by treating the ISA with different mineral acids and bases. The work reported by these authors suggested ISA as a resource material for zeolites and ferrous particles, and a potential material for silica synthesis.

In our earlier reported work, it was found that Indian ISA has a large amount of Ca, Mg, Si, Al, and Fe oxides. The majority of sources of such minerals in ISA is coal or charcoal powder, or any other burning agent mixed for the smoother burning of incense sticks [1,9]. Therefore, Yadav et al. concluded that ISA reflects the elemental composition of coal fly ash (CFA), which mainly contains silica, alumina, ferrous oxides (Fe_3_O_4_, γ-Fe_2_O_3_, β-Fe_2_O_3_), and some other trace element oxides [12,13]. Both of these ashes have Si, Al, and Fe, along with traces of Na, P, K, Ti, etc. However, there is a major difference in the content of carbon, which is much higher in ISA since it is totally organic in nature, while CFA is 40–60% silica, with ISA only 15–20% silica. Besides this, ISA is more than 50% Ca and Mg oxides while CFA has only 5–15%, depending on the source of the coal and the class of CFA. Furthermore, CFA is 15–25% carbon, including both burned and unburned, and also contains numerous metal oxides in trace amounts [14]. These minerals from ISA can be extracted directly in their original form at the nano- or micron-sizes, or they may be processed further for the recovery of respective elemental oxides [15].

ISA is about 5–8% ferrous minerals, which is the 4th highest fraction of minerals present in ISA, as confirmed by XRF [3]. The magnetic fractions can be preferably extracted from ISA using both dry and wet magnetic separation methods. Recently, Gupta et al. reported the recovery of ferrous fractions from ISA by dry and wet magnetic separation methods, and concluded that wet method-based ferrous recovery is more efficient [16]. Further, Yadav et al. used these ISA-extracted ferrous particles for the synthesis of amorphous iron oxide nanoparticles. Recently, Yadav et al. used sonochemically-synthesized amorphous iron oxide nanoparticles for the removal of Congo red dye from wastewater. Calcium oxide is also a major component of ISA, and can be extracted by one of the reported techniques available for the recovery of calcium oxides from calcium-rich waste such as gypsum [17,18], eggshell waste [19], mollusks [20], cockle eggshell waste, etc. [21]. Calcium oxide is an exceptionally important industrial compound, as it is used as a catalyst in numerous chemical reactions, for biodiesel production [22], as non-toxic remediable particle, as an additive in refractory material, in paints, and also as a nano adsorbent [23]. CaO has various applications in biodiesel production, tissue engineering, biosensors, power production, and the cement and petroleum industries [24].

The alumina content in ISA can vary from 4–5%, along with several impurities. The source of alumina in ISA is coal powder only, which is used as a facilitating agent for the burning of incense sticks. Alumina micro- and nanoparticles find applications in environmental clean-ups [25], ceramics [26], adsorbents [27], catalysis [28], and insulating and fire-proof materials [24,29]. Generally, alumina micro- and nanoparticles exhibit transition and metastable phases [30]. Among these metastable forms, gamma (γ) alumina has applications in optoelectronics [31], metallurgy, spacecraft materials, ceramics, and glasses as an adsorbent and a coating material, and in the automobile and petroleum industries as a catalyst and catalyst support [32,33,34,35,36].

The silica present in ISA can be recovered by one of the various techniques applied for the recovery of silica dioxides from silica-rich wastes such as fly ash [37,38], rice husk ash [39], and red mud [40]. Biocompatible, bioconjugated, and doped silica nanoparticles are widely used in cancer cell imaging [41,42], DNA and microarray detection, barcode tag separation, drug delivery, and the purification of biological molecules and cells [37,38,43]. Silica nanoparticles are used in molecular sieves, resins, silica-based catalysts, and various other materials [38].

Assadi and Sahajwalla (2014), reported alternative sources of carbon. They reported on the recycling of carbon for the steel industry. By using simulations, they reported that they reached up to 41% dissolution of polycarbonate carbon content, which is comparable to the dissolution of C from graphite, which reaches up to 58%. Finally, they exhibited that polycarbonate H content could not dissolve in molten Fe, but rather escapes in gaseous form. Therefore, they reached the conclusion that waste polycarbonate makes a feasible C source for steelmaking [44]. Sahajwalla et al. recycled end-of-life polymers in an electric arc furnace steelmaking process. Their study established fundamentals of the interaction of waste polymers with slag and metal in steelmaking methods [45].

Once all the value-added minerals are recovered from ISA, then the final residue left after silica extraction using a strong base may become reactive, and could pose a potential threat to the environment. Therefore, here, it will be transformed into a zeolitic material by processing with acid or bases, so that it may find applications in industries. Recently, Yadav et al. reported the synthesis of Ca-rich zeolites from incense stick ash, which they used for the remediation of dye from wastewater. They synthesized gismondine zeolite sized was in microns. Several other works have also supported the use of ISA as a material for the synthesis of zeolites [46].

Here, we have proposed various methods for the recovery of all the possible value-added minerals from ISA by chemical approaches, and for transforming the final ISA into a non-hazardous material. ISA was collected from temples and initial analysis was done for the identification of major and minor elemental composition and morphological features. It was processed in sequential order for the recovery of magnetic fractions, calcium oxides, silica, an alumina, and was then finally processed into a nonhazardous zeolite product. The confirmation of purity and formation of the value-added minerals from ISA were analyzed with sophisticated instruments. The utilization of ISA in metallurgy will definitely minimize solid waste, and allow ISA to serve as an economical waste material for the recovery of ferrous materials and silica, and to serve other purposes in ceramics industries [3].

## 2. Materials and Methods

### 2.1. Materials

Materials included incense stick ash, sieves of different screen sizes, 100 mL beakers (2–3), 100 mL round bottom flasks (2–3), double-distilled water (ddw), magnetic rods (Axiva), neodymium magnets (cylindrical shape, procured from A-Z Magnet, Chandni Chowk, Delhi, India), concentrated HCl (RENKEM, Gujarat, India, sodium hydroxide pellets (Hi-media, Gujarat, India, methanol (SRL, Gujarat, India), concentrated H_2_SO_4_ (RENKEM), ethanol (Sigma Aldrich, Hamburg, Germany), and Na_2_CO_3_ (SRL, Gujarat, India).

### 2.2. Methods

(1) Collection and processing of incense stick ash

**Figure 1:**
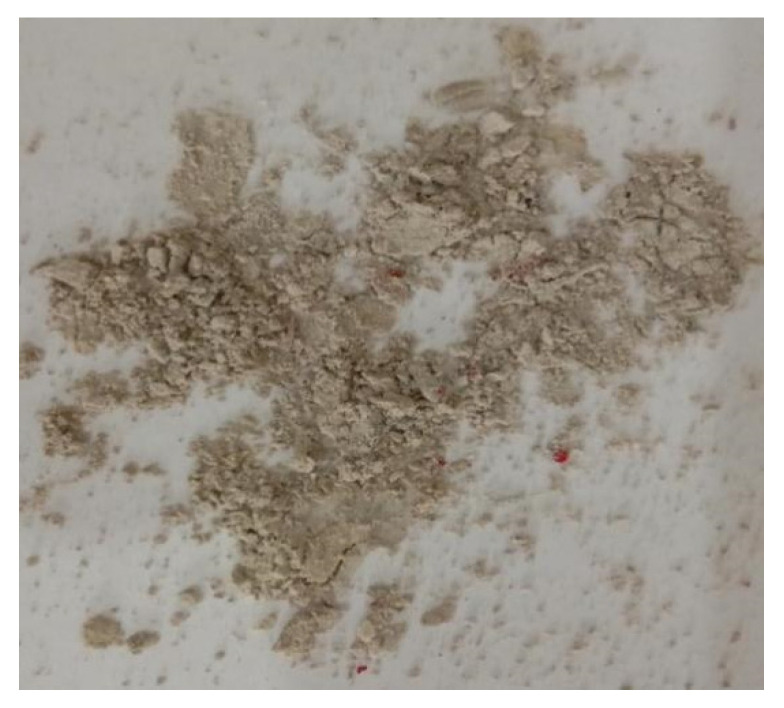
Photograph of incense stick ash collected from a temple.

Incense stick ash (ISA) shown in Figure 1, was collected from the local temples situated in Gandhinagar, Gujarat, India. Further it was sieved with a sieve set in order to remove large particles and unburned bamboo and incense sticks. The sieved ISA was dried in an oven at 60–70 °C overnight to remove moisture and then stored in air-tight sample vials. Then, the fine ISA was analyzed by X-ray fluorescence spectroscopy (XRF) and field emission scanning electron microscopy with electron diffraction spectroscopy (FESEM-EDS) to find the elemental composition and morphology of ISA.

(2) Extraction of magnetic fractions from ISA

A slurry of ISA and distilled water was prepared at a 1:5 ratio in a 200 mL beaker. Furthermore, a magnetic bead of 4 cm in length and 1 cm in width was added to the ISA slurry. The slurry was kept on a magnetic stirrer, and mild stirring was done at 300–500 rpm. Due to magnetic stirring, the ferrous particles adhered to the magnetic bead, which was taken out and the attached magnetic fractions removed mechanically.

**Figure 2:**
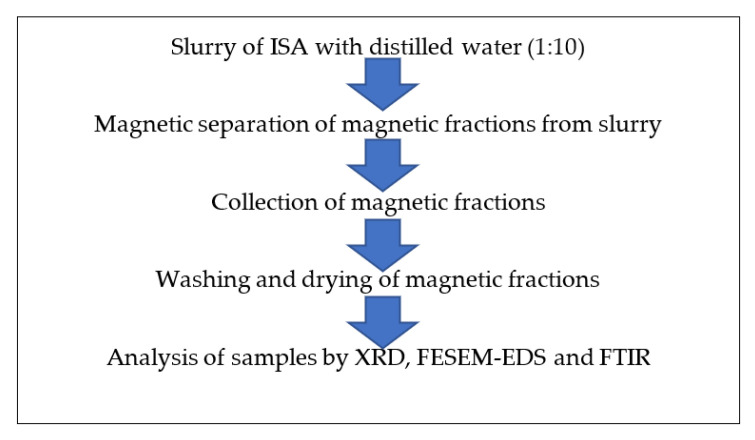
Schematic extraction of magnetic fractions from ISA slurry.

The bead was again added to the beaker with slurry and stirring was performed. The attached ferrous fraction was again mechanically removed from the bead. This process was repeated several times until a sufficient quantity of magnetic fractions were collected. Then, the collected magnetic fractions were placed on the magnetic stirrer with mild stirring without a bead. The magnetic ferrous fraction adhered to the center, while the impurities present at the periphery of the Petri plate were removed by a cotton bud. This process was also repeated several times until no more non-magnetic fractions as impurities were observed. The obtained ferrous fraction was dried on the hot plate at 40–50 °C and further characterized by instruments for purity, morphology, and other properties. Figure 2 shows the schematic extraction of magnetic fractions from ISA.

(3) Synthesis of alumina particles from non-magnetic fractions of ISA

The non-magnetic fractions that remained after the separation of magnetic particles were dried to complete dryness in an oven at 50–60 °C. About 6 g of ISA residue was treated with 30 mL of 0.5 M HCl for 1–2 h on a magnetic stirrer at 400–700 rpm at 70–90 °C. On the completion of the reaction, the mixture was allowed to cool at room temperature (RT) and the reaction product was then centrifuged for 10–15 min at 5000 rpm. About 10 mL of liquor was carbonated in a round bottom flask by adding CO_2_ at a rate of 5 vvm.

**Figure 3:**
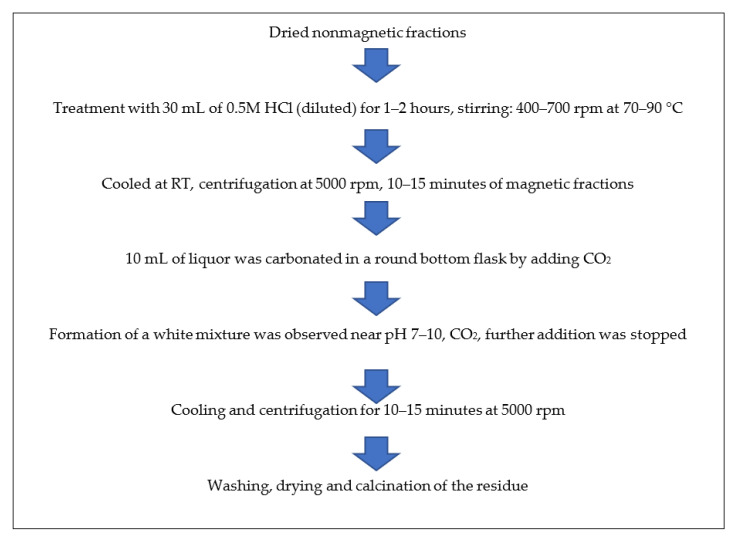
Schematic method for synthesis of alumina particles from non-magnetic fractions of ISA.

The formation of a white mixture was observed near pH 7–10, after which carbonation was stopped. The resultant mixture was allowed to cool and centrifuged for 10–15 min at 5000 rpm. Then, the obtained liquor was discarded and the precipitate was retained. The precipitate was washed many times with double-distilled water and ethanol and then dried in an oven at 50–60 °C overnight. Finally, the white powder was dried and calcined in a muffle furnace at 600 °C for 4 h and analyzed by various advanced instruments for confirmation of the obtained particles. Figure 3 shows the schematic methods for the synthesis of alumina particles from ISA residue.

(4) Synthesis of silica nanoparticles from ISA

**Figure 4:**
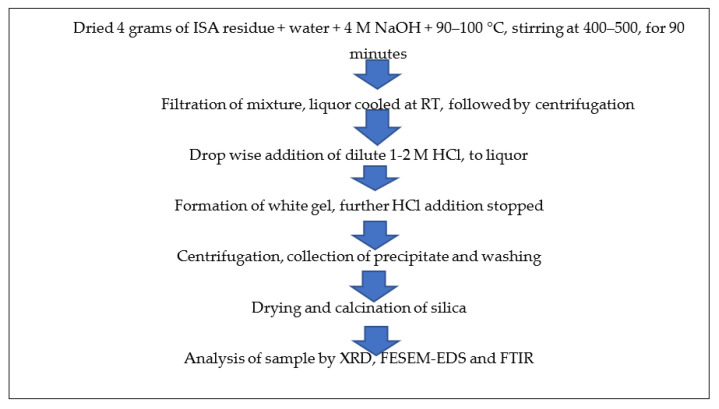
Schematic method for synthesis of silica nanoparticles from non-magnetic fractions of ISA.

About 4 g of non-ferrous incense ash residue after extracting alumina (rich in silica) was added to 4 M NaOH at a ratio of 1:5 and a temperature of 90–100 °C, but most optimally at 95 °C, along with continuous stirring at about 400–500 rpm for 60–90 min in a round bottom flask with a reflux condenser. After the completion of the reaction, the mixture was filtered by Whatman filter no. 42; the filtrate was retained while the residue was washed and dried for future use. Then, 10 mL of extracted filtrate, i.e., sodium silicate, was taken in a 100 mL beaker and neutralized with the dropwise addition of diluted 1–2 M HCl from the burette. The beaker was continuously rotated to mix the contents properly. After some time, a white gel began to form near pH 7–10, after which HCl addition was stopped. The beaker was covered with aluminum foil and the silica gel was allowed to age for 24 h at room temperature. After 24 h of ageing, the mixture was centrifuged at 5000–7000 rpm for 5–10 min. The liquor was decanted and the precipitate was retained. The precipitate was washed 2–3 times with warmed double-distilled water and ethanol to remove any impurities in the form of acids or salts. The precipitate was dried in an oven at 40–60 °C overnight and calcined at 400 °C in a muffle furnace for four hours. Figure 4 shows the schematic method for the synthesis of silica nanoparticles from ISA residue.

(5) Synthesis of CaO from ISA

The ferrous free incense stick ash (FFISA) was mixed with 2 N HCl (solid-to-liquid ratio of 1:6) with continuous stirring at room temperature for 2–3 h in a round bottom flask. After the completion of the reaction, the mixture was centrifuged at 5000–7000 rpm for 10–15 min at room temperature (RT), after which the liquor was decanted while the residue was dried overnight in an oven at 40–50 °C. The residue was mixed with 4 N H_2_SO_4_ at a 1:5 ratio and a temperature of 80–100 °C for 60–90 min, with continuous stirring at 300–400 rpm in a round bottom flask with a reflux condenser. The mixture was centrifuged at 5000–7000 rpm for 5–10 min at room temperature to obtain the residue, which was washed 2–3 times with ddH_2_O and dried in an oven at 60 °C for 5–6 h. The residue was treated with 4 M NaOH, with a solid-to-liquid ratio of 1:5 at 90–95 °C for 60–90 min, with continuous stirring at 300–400 rpm in a round bottom flask with a reflux condenser. After the reaction time was over, the mixture was centrifuged at 5000–7000 rpm for 5–10 min at room temperature to obtain the solid fraction, which was washed several times with ddH_2_O and dried in an oven at 40–50 °C until complete dryness. The residue was calcined at 450 °C for three hours in a muffle furnace. Finally, it was analyzed by various instruments for detailed information and confirmation of the formation of particles. Figure 4 shows the schematic synthesis of calcium oxide particles from ISA residue.

(6) Utilization of final ISA residue

**Figure 5:**
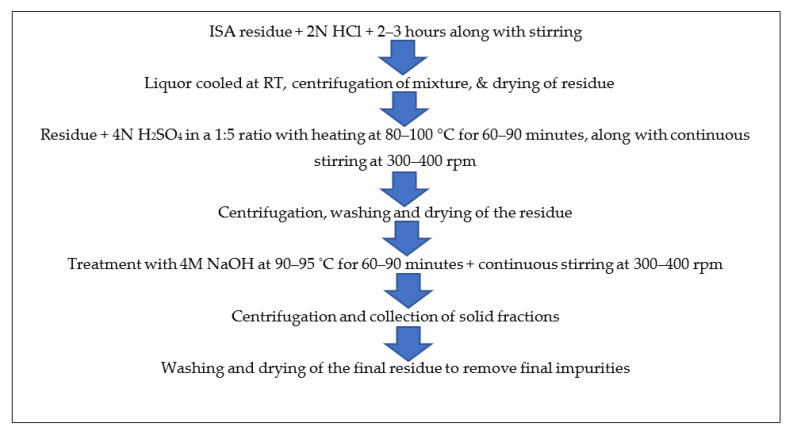
Schematic method for synthesis of calcium oxides from non-magnetic fractions of ISA.

The final residue obtained after the treatment of ISA with NaOH during silica extraction was washed 3–4 times with distilled water in order to remove any alkali moieties present on the surface of the ISA residue. Then, the residue was dried in an oven at 40–50 °C to remove water. Finally, it was calcined at 400 °C for 4 h in a muffle furnace before analysis. The steps involved in the processing of ISA residue for the synthesis of calcium oxides from the Ca-rich leachate is shown in Figure 5.

## 3. Characterization

The ISA and all the recovered and synthesized value-added materials were analyzed by Fourier-transform infrared spectroscopy (FT-IR) of Perkin-Elmer (USA) SP-65 make, particle size analyzer (PSA), X-ray diffraction (XRD), field emission scanning electron microscopy with electron diffraction spectroscopy (FESEM-EDS), and transmission electron microscopy (TEM). For the chemical composition of ISA, the major oxides were generally analyzed by XRF using a Horiba, Japan model no. XGT-2700 X-ray analytical microscope fitted with a high purity silicon detector (XEROPHY) X-ray tube with an Rh target. The analysis was done to detect the chemical content of the ISA sample. About 5–8 g of ISA was mixed with NaBr powder and a pellet was prepared by pellet making machine. The PSA is based on the laser scattering technique; the particle size distribution and surface charge of the particle were analyzed by a Malvern Zetasizer S90 (Malvern, UK). The extracted particles were dispersed in double-distilled water and sonicated for 10 min in an ultrasonicator (Sonar, 40 Khz) before analysis. FTIR spectroscopy measurements of all the powder samples were analyzed by preparing the KBr pellet technique by using a Perkin-Elmer (Waltham, MA, USA) SP-65 at a resolution of 2 cm^−1^. This was used for the detection of various functional groups and ultimately, the minerals present in the recovered samples. The XRD analysis was done for the phase identification of various amorphous and crystalline minerals present in the samples. The XRD patterns were recorded using a Bruker Advance D-8 (Karlsruhe, Germany) instrument equipped accelerometer in the 2θ range of 20–70° with a step size of 0.02 and a time of 5 s per step at 40 kV voltage and a current of 30 mA. The surface shape and size analysis of the samples was done by FEI Nova NanoSEM (FEI, Hillsboro, OR, USA) FESEM at variable magnification. The analysis was carried out by spreading the powder samples on double-sided carbon tape, and gold sputtering was performed for 10 min. The elemental composition of all the sample particles was investigated with the attached Bruker-made EDS analyzer.

## 4. Results and Discussions

### 4.1. Chemical and Elemental Properties of ISA by XRF

The elemental composition of typical Indian ISA is given below in Table 1 and Table 2, with Table 1 showing elemental oxides as found by XRF, and Table 2 showing trace amounts of heavy metals as found by ICP-OES. Indian ISA is 50–60% calcium and magnesium oxides, 15–25% carbon, 15–20% silica, and 5–8% iron oxides and alumina, while the remaining is Na, P, Ti, etc. ISA also have several toxic heavy metals such as Cu, Cr, Ni, Co, Zn, Mo, Hg, As, and Pb. Since all these metal oxides and heavy metals are present in coal and its fly ash, the black-colored incense sticks using coal powder exhibit almost identical content to CFA [9,13].

### 4.2. Morphological Analysis of ISA by FESEM & XRD

**Figure 6:**
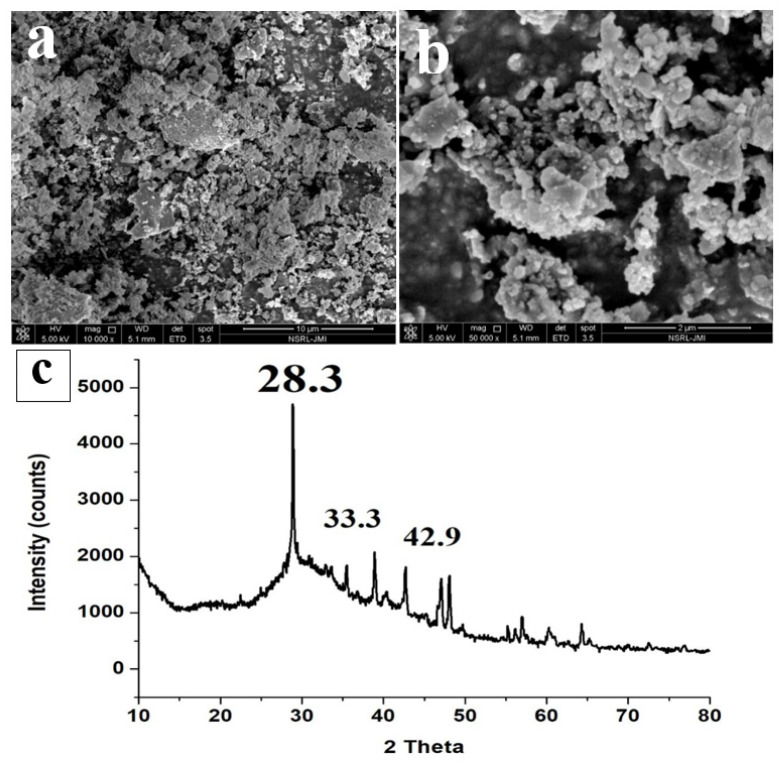
FESEM micrographs (**a**,**b**) and XRD (**c**) of ISA.

The FESEM image (Figure 6a,b) of ISA shows that the particles are highly aggregated and gather together to form lumps. The ISA particles are generally irregular in shape, with sizes varying from 300 nm to several microns [9]. Some of the smaller particles appear to be particles lying on top of other particles, giving a final cylindrical shape structure. The size of ISA is reduced after burning; this could be due to the transformation of organic molecules during burning, or it could also be due to the passing of ISA through sieves, which might have separated the larger particles. Figure 6c presents a typical XRD pattern of ISA, which shows major peaks at 2 theta, 28.3, 33.3, 37.4, 42.92, and 46.5. The sharp peak at 28.3 is attributed to the quartz and mullite peak formed by the crystalline phase of ISA. A small peak at 32–35° is attributed to the ferrous oxides, mainly magnetite and hematite. The peak at 42.92° is due to the calcite phase of the ISA, which again is crystalline in nature. This phase is the most dominant in the ISA, as evident by the XRF data.

Table 3 clearly indicates the higher composition of O and Si, while Fe is present as an intermediate element in the ISA. The focused region may be rich in Fe. There are also traces of Al, K, Ti, Mg, C, and Ca. ISA is rich in C and Ca, but here their composition is very low. This could be due to the EDS focusing on the Fe-rich area, rather than the Ca and C rich-area. Generally, ISA has higher C and Ca due to its organic source of carbon [14].

### 4.3. Results and Discussion of Magnetic Fractions

ISA is a mixture of various minerals such as calcite, silicates, periclase or magnesia, quartz, etc., which come from coal powder [2]. All these minerals are valuable materials that can be used for numerous applications. The ferrous particles come from coal powder, which constitutes 5–8% of ISA [13]. Such ferrous particles can be analyzed by various instruments for detailed mineralogical, elemental, and morphological properties.

**Figure 7:**
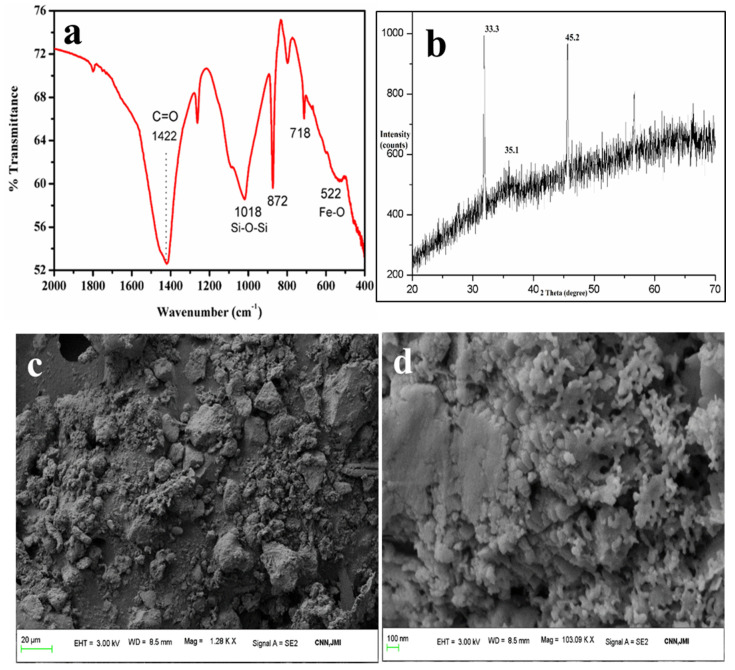
Analysis of iron oxides: (**a**) FTIR, (**b**) XRD, (**c**,**d**) FESEM micrographs.

The FTIR spectra (Figure 7a) show peaks at 522 cm^−1^, 718 cm^−1^, and 872 cm^−1^, which are attributable to Fe-O, and another peak at 1089 cm^−1^ that is assigned to the Si-O-Si bonds of silicates or quartz [47]. This indicates the presence of quartz with the ferrous particles. The band at 872 cm^−1^ [48,49] and 718 cm^−1^ could also be due to the external plane-bending vibrations of carbonates and the internal plane-bending vibrations of O-C-O of carbonates, respectively [50,51]. The band at 1422 cm^−1^ is attributable to the asymmetric vibrations of the C-O bond in the carbonate [52]. This confirms the association of calcium carbonates with ferrous particles as a remarkable impurity, which was also confirmed by the FESEM-EDS. Similar results were also obtained by Yadav et al., in which they extracted ferrous fractions from ISA [16].

The XRD pattern (Figure 7b) also reveals strong intense peaks at 33° and 35°, which are due to hematite and magnetite phases present in the ferrous samples [53]. A small peak near 24° and 27° is due to the quartz form and amorphous silica associated with the ferrous particles. Besides these, there were no other peaks found in the sample. Table 4 shows the elemental composition of the iron oxide particles synthesized from ISA.

The higher wt. percentages of Fe (54.98%) and O (30.61) indicate the formation of iron oxide particles from ISA with high purity. Since ISA has a large amount of C, it also remained in the final sample. Besides, there were traces of Si and Al, as both these elements were present in the original sample, i.e., ISA.

The FESEM micrographs (Figure 7c,d) showed sphere- to irregular-shaped iron oxide particles. The particles were observed to be highly agglomerated in the form of clusters, with a size of less than 100 nm. Similar results were also reported by Yadav et al. who found that ferrous particle size varied from 80 nm to several microns due to the aggregation of the ferrous particles [16]. The spectra obtained from EDS exhibited sharp peaks for Fe and O as major elements, along with peaks for other elements like Al, Si, Mg, Na, Ca, K, P, Ti, and C (data not shown here). Similar results were also obtained by Yadav et al., who found high carbon and Ca content [54]. The final red color iron oxide pigment was analyzed for purity by the FESEM-EDS. This analysis revealed that the purity of the sample was about 60–80%, with major maghemite phases, as also confirmed by XRD.

Indian ISA is 4–6% alumina, as revealed in the XRF data. The source of aluminates in the ISA is the coal powder used as a facilitator for the smooth burning of incense sticks. In coal, alumina is present in the form of aluminosilicates, sillimanite, or mullite, mainly in crystalline forms. Aluminates are also always associated with other elements, such as Ca, Si, and Na, which are present in coal.

The Al present in the ISA was treated with diluted 1–2 M HCl to leach the Al from the alumina of ISA, which was further used as a precursor material for the synthesis of alumina via the precipitation method using ammonium hydroxide as a precipitating agent. Here, Al reacts with HCl to form AlCl_3_, and when it undergoes carbonation, it forms aluminum hydroxide. The calcination of aluminum hydroxide at 600 °C for 4 h ensured the removal of water and moisture. Furthermore, it was analyzed by the sophisticated instruments for detailed analysis of the synthesized powder.

**Figure 8:**
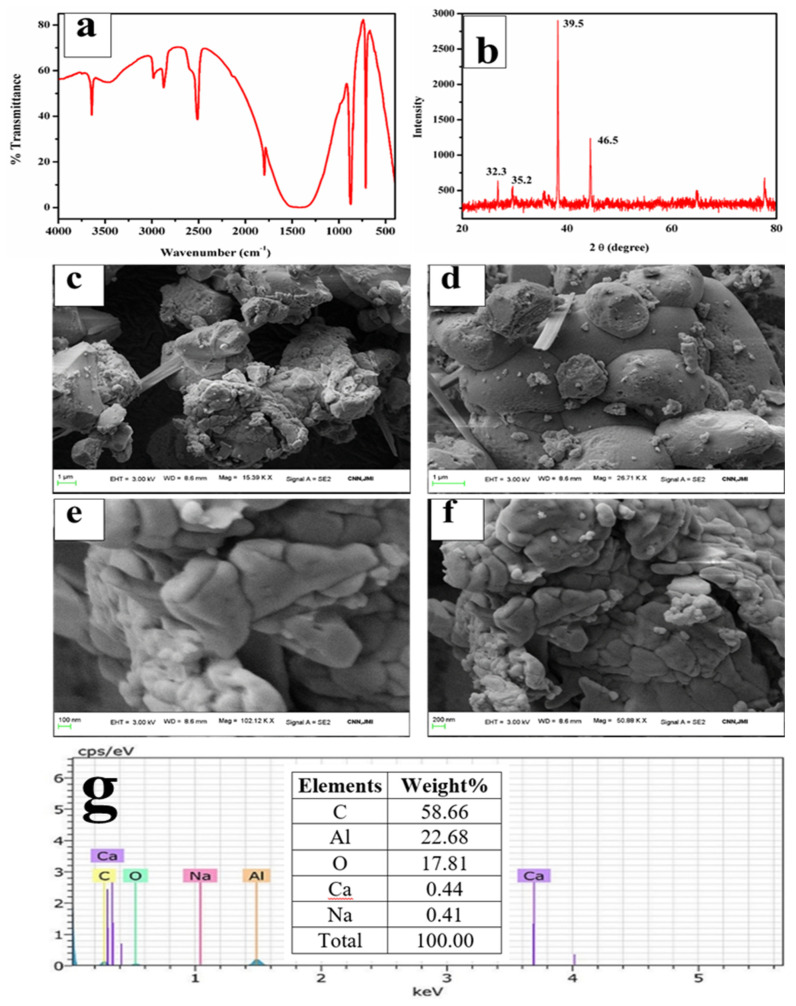
Analysis of alumina microparticles: (**a**) FTIR, (**b**) XRD, (**c**–**f**) FESEM micrographs, (**g**) EDS spectra.

The FTIR spectra in Figure 8a exhibited bands around 448 cm^−1^ and 717 cm^−1^, which were assigned to the stretching of Al-O (AlO_6_) bands, and the bands at 865 cm^−1^ were assigned to the bending vibrations of Al-O bonds (AlO_6_) [55,56]. A broad band in the region of 1200–1500 cm^−1^ with a peak centered at 1438 cm^−1^ is attributable to the carbon molecules present in the sample, which are impurities in the ISA [57]. The band at 1438 cm^−1^ is due to the C=O, confirming the association of carbon with the synthesized alumina, which may come from ISA. The band at 1438 cm^−1^ could also be attributed to the formation of the boehmite phase of alumina [58,59]. The band around 2509 cm^−1^ is attributable to the adsorption of atmospheric carbon on the alumina [60]. The broad bands around 3064 cm^−1^ and 1438 cm^−1^ were assigned to the combination of asymmetric stretching vibrations of Al-OH (boehmite) [61] and the stretching and bending modes of adsorbed water [62]. This indicates that the alumina product is a hydrate.

The XRD analysis of alumina particles in Figure 8b reveals peaks at 32.3°, 35.5°, 39.5°, and 46.5°, which indicate the presence of mixed phases of alumina oxides, i.e., boehmite, gamma-alumina, delta-alumina, and aluminum sulfate [63,64]. The peaks at 32.3° and 35.5° are due to the aluminum hydroxides [65]. There is a major and sharp peak at two theta 39.5°. Nevertheless, since calcination was done at 600 °C, the mixed phases of boehmite, gamma-alumina, delta-alumina, aluminum hydroxide, and aluminum chloride were also observed. The gamma-alumina has a small intensity peak at 46.5°, which supports previous work done by Hosseini et al. [66].

The FESEM micrographs of alumina microparticles in Figure 8c–f showed cuboidal- to irregular-shaped particles sized 0.1 to several microns, which aggregated together to form a lump. The shape and size of the particles are similar to the results obtained by Ghosh et al. [67]. The alumina particles were spherical and highly aggregated, which is due to the eggshell. The EDS spectra show sharp peaks for Al, O, and carbon; excepting these, there are no other elements in Figure 8g that were present initially in the ISA.

### 4.4. Synthesis and Characterization of CaO & CaCO_3_ from ISA

Calcium carbonate has generally three different crystalline phases or polymorphs that co-exist in nature.

One is calcite, which is thermodynamically stable under ambient conditions [68]. The second is aragonite—a high-pressure polymorph that is less stable than the calcite [69]—and the third is vaterite, which is the least stable among all the three and which has a tendency to transform into the other two polymorphs [70,71].

**Figure 9:**
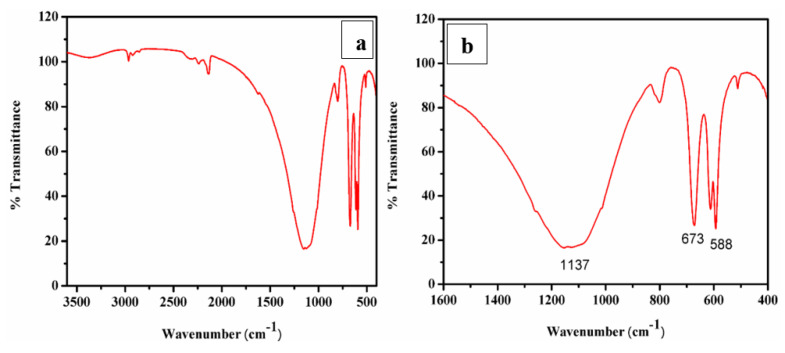

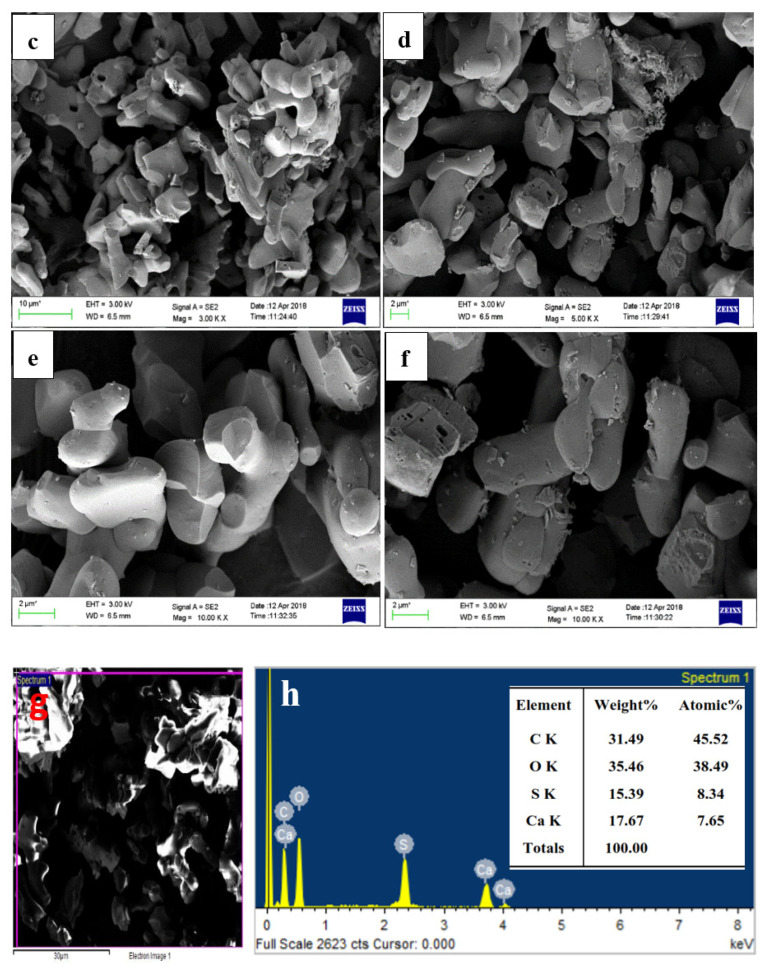
(**a**,**b**) FTIR and deconvoluted, respectively.; (**c**–**f**) FESEM images; (**g**) EDS spot; (**h**) EDS spectra.

A typical FTIR spectrum of CaO derived from ISA is shown in Figure 9a,b. The minor band near 3400 cm^−1^ in the spectra was attributed to the (-OH) stretching vibration and bands near 2200–2400 cm^−1^ were attributed to the adsorbed atmospheric moisture and carbon dioxide and the formation of Ca(OH)_2_. As the CaO molecule is highly hygroscopic, it absorbs water very quickly [72]. The band at 2509 cm^−1^ is due to the carbonate peak [73] and a small band at 3654 cm^−1^ is due to the OH molecule. The broad band around 1400–1498 cm^−1^ indicates a symmetric stretching vibration due to the carbonation of CaO by the adsorption of atmospheric CO_2_. A small band at 870 cm^−1^ demonstrates the presence of carbonate species, and a tiny band at 560 cm^−1^ indicates vibrations of the Ca-O bond. The broad band with a peak centered at 1137 cm^−1^ indicates a symmetric stretching vibration due to the carbonation of CaO by the adsorption of atmospheric CO_2_ [74]. The small band at 865 cm^−1^ was attributed to the symmetric stretching vibration of the C-O bond and the presence of carbonate species [75].

A sharp band at 673 cm^−1^ was attributed to the external plane bending vibrations of the CO_3_^2−^ [76]. Another sharp band at 588 cm^−1^ was attributed to the vibrations of the Ca-O bond [77]. A sharp peak in the FTIR revealed that the synthesized particles are polycrystalline in nature. The FTIR peaks confirmed the extraction and synthesis of CaO/CaCO_3_ from incense stick ash by showing the characteristic peaks of carbonate.

The FESEM micrographs from Figure 9c–f reveal rod and dumbbell-shaped particles from 0.1 to several microns, which aggregated together to form a lump. The shape and size of the particles are similar to the results obtained by Ashok et al. and Jirimali et al. [78]. In both cases, the synthesized calcium oxide particles were spherical to rod-shaped [79] and aggregated, with sizes varying from 500 nm to several microns [78]. The EDS spot (Figure 9g) spectra in Figure 9h shows the sharp and major peaks for Ca, O, and C as major elements, confirming the formation of both calcium oxide and calcium carbonate. Besides this, there is a prominent peak for sulphur as an impurity from the sulphuric acid treatment due to improper washing. The calcination at higher temperatures might have eliminated the sulphur content in the synthesized calcium carbonate particles.

### 4.5. Synthesis and Characterization of Nanosilica from ISA

**Figure 10:**
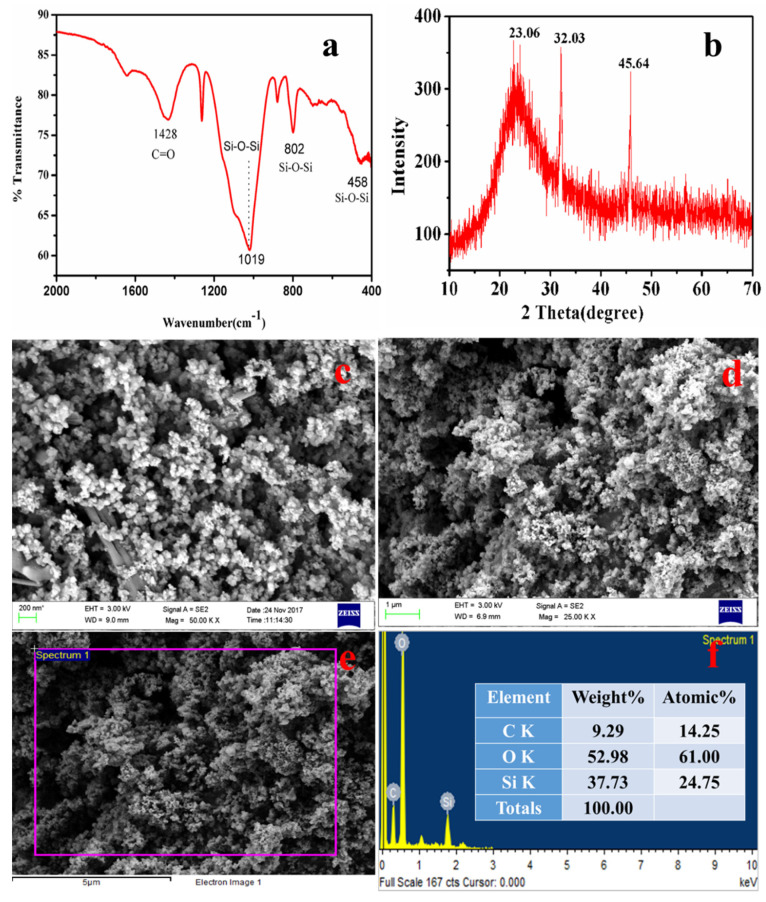
Analysis of silica nanoparticles from ISA: (**a**) FTIR, (**b**) XRD pattern, (**c**,**d**) FESEM images, (**e**) EDS spot, and (**f**) EDS spectra.

The typical FTIR spectra of nanosilica is shown in Figure 10a, with characteristic bands at 458 cm^−1^, 802 cm^−1^, and 1019 cm^−1^ [37,38]. A sharp band at 458 cm^−1^ was assigned to the bending vibrations of Si-O-Si [80], while the band at 802 cm^−1^ indicates the symmetric vibrations of Si-O-Si [81], and a broad band at 1105 cm^−1^ is due to the asymmetric vibrations of Si-O-Si of silica [37]. The band at 1428 cm^−1^ was attributed to the C=O bonds present in the sample, which might be from the carbon of the ISA. ISA has numerous organic and inorganic carbon compounds that come from the fragrances used for aroma. Moreover, this could also be due to the unburnt bamboo sticks. The synthesized nanosilica particles were pure and amorphous in nature.

Figure 10b presents a typical XRD pattern of nanosilica, which reveals a broad hump starting at 15° and terminates at 30°, with a peak centered at 23.06°. This confirmed the amorphous nature of the nanosilica, while the broadness of the hump revealed that the synthesized nanosilica at the nanoscale [82]. Similar crystalline peaks for amorphous silica was also observed by the Shadab Ali khan et al. in the biosynthesis of silica nanoparticles from coal fly ash [83].

The FESEM micrographs of silica nanoparticles in Figure 10c show that there are two types of particles. The rod-shaped larger particles are mainly sized is in microns, while the other smaller particles are mainly spherically shaped. The smaller spherically shaped particles aggregated to form a floral-shaped structure. Figure 10d shows an image of nanosilica after the purification step, i.e., after treatment with 1–2 M HCl for 90 min at 110 °C followed by calcination at 400 °C for four hours. Therefore, the purified nanosilica is mainly dominated by the spherical particles, whereas the larges particles, mainly impurities, were dissolved or broken down. After purification, the size of the individual nanosilica particles ranges from 40–80 nm, but as these particles are naked and smaller, they show aggregation. The particles aggregated and formed a cluster-type structure, indicating that this aggregation of particles was due to the calcination temperature. Yadav and Fulekar (2019) and Deshpande and Joshi (2014) also obtained a cluster-like structure of silica particles from coal fly ash (CFA) [37].

Figure 10e is an EDS spot, while Figure 10f is the EDS spectrum of the nanosilica confirming the presence of Si and O, indicating the formation of the nanosilica. Before purification and calcination, there were impurities in the form of organic carbon, Na, Cl, Al, Mg, etc. which after the purification step were reduced drastically. The Al, Na, K, and Mg, being acid-soluble, were eliminated, while the organic carbon was reduced due to calcination at high temperature, Therefore, the final nanosilica was purified many times after the purification step. The Si content was 37.7% by weight, whereas O content was 52.9% by weight. The remaining portion is carbon, which cannot be reduced further as its initial content was very high in the ISA. While the organic carbon was eliminated during calcination, the inorganic carbon was still present with the nanosilica. The presence of Si and O in high amounts confirms the formation of nanosilica from the ISA non-ferrous residual material.

### 4.6. Analysis of Final ISA Residue (FISAR)

The final residue after the extraction of ferrous minerals, calcium oxides, alumina, and silica from ISA was analyzed for various changes in its chemical and morphological properties. The final residue was analyzed by FESEM-EDS for its elemental composition and morphological changes.

**Figure 11:**
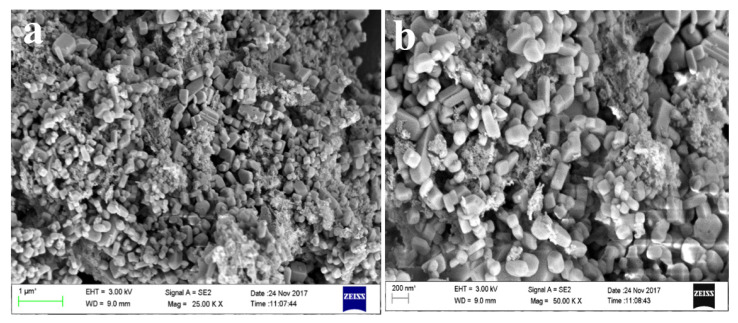
(**a**) FESEM micrograph of FISAR and (**b**) FESEM micrograph of gismondine zeolite adopted from Yadav et al. [84].

The FESEM micrograph of FISAR in Figure 11a reveals the cuboid- and rhombohedron-shaped non-aggregated particles. The nanoparticles observed in the FESEM images are cuboid-shaped, while few are long rods. Short cuboidal particles were more common than long ones. The range of nanoparticles varied lengthwise from 200–600 nm, and widthwise from 100–300 nm. The EDS analysis showed the existence of Ca, C, O, Al, S, and Si elements in the sample; the existence of Si and Al along with the high group II alkali metals, i.e., Ca, indicates the possibility of utilizing the residue for zeolite synthesis. The results were in agreement with Yadav et al., as shown in Figure 11b [84].

Yadav et al. also reported the synthesis of Ca-rich zeolites from ISA, which were cuboidal in shape and had sizes varying from 200 to 700 nm. After analysis, it was found that the synthesized zeolites belong to the class gismondine (Ca_2_Al_4_Si_4_O_16_._9_H_2_O), and the zeolites were later applied for the remediation of dye samples from wastewater. The chemical composition of the synthesized zeolites confirmed the formation of the particles; the whole elemental composition is given in Table 5. The characterization of the zeolites revealed similar properties to the final residues obtained in our experiment.

### 4.7. Conclusions

The extraction of ferrous minerals, calcium oxide, alumina, and silica from ISA is not only a green method, but also opens a new horizon in the field of ISA utilization and minimization of water-based pollution. The comprehensive characterization of ISA particles revealed the micron-sized irregular shape of the particles; XRF and EDS analysis confirmed that the ash was Ca-rich along with containing carbon and Si. The ferrous particles extracted from the wet slurry of the ISA were sized below 100 nm, but aggregated to form clusters. The shape of the particles was irregular to spherical; the difference in shape could be due to the irregular combustion temperatures of incense sticks. The as-synthesized calcium oxide/calcium carbonate particles were confirmed by FESEM, with sizes of 1–5 microns and spherical and rod shapes, along with a highly aggregated nature. The nanosilica was spherical in shape and aggregated together to form a network similar to a floral shape. The size of which was 60–120 nm, as confirmed by FESEM. The final residue revealed similarity with the low Si-containing zeolites. In the future, all these value-added minerals may prove to be alternative sources of ferrous, calcium, silica, alumina, and zeolites for industries, with numerous applications. The synthesis and recovery of such value-added minerals from waste incense stick ash reduces solid waste, as well as water pollution.

## Figures and Tables

**Table 1:** Elemental composition of incense stick ash as found by XRF.

Elements	Wt%
CaO	49.2
SiO_2_	20.55
Al_2_O_3_	4.78
MgO	4.0
K_2_O	8.23
Fe_2_O_3_	4.288
SO_3_	4.45
P_2_O_5_	4.5

**Table 2:** Concentration of heavy metals in ISA as found by ICP-OES.

Elements	mg/L (ppm)
Cr	1.8
Cd	0.002
Co	0.31
Cu	3.5
Ni	1.284
Pb	0.156
Zn	2.825

**Table 3:** Elemental composition of ISA by EDS adopted from Yadav et al. [14].

Element	Weight%
O	41.33
Al	1.12
Si	10.82
C	1.1
Fe	3.5
K	0.73
Ti	1.02
Mg	0.13
Ca	0.24
Total	100.00

**Table 4:** Elemental composition of synthesized iron oxide particles as found by EDS.

Element	Weight%
C	12.55
O	30.61
Al	0.56
Si	1.30
Fe	54.98
Totals	100.00

**Table 5:** Elemental composition of Ca-rich zeolites synthesized from ISA.

Element	Weight%
C	9.84
O	45.57
Al	0.66
Si	4.19
S	18.31
Ca	21.52
Total	100.000

## Data Availability

Not Applicable.
